# The Opportunities and Challenges of the First Three Years of Open Up, an Online Text-Based Counselling Service for Youth and Young Adults

**DOI:** 10.3390/ijerph182413194

**Published:** 2021-12-14

**Authors:** Paul Siu Fai Yip, Wai-Leung Chan, Christian S. Chan, Lihong He, Yucan Xu, Evangeline Chan, Yui Chi Chau, Qijin Cheng, Siu-Hung Cheng, Florence Cheung, James Chow, Shirley Chow, Jerry Fung, Siu-Man Hsu, Yik Wa Law, Billie Lo, Sze-Man Miu, Wai Man Ng, Ken Ngai, Christy Tsang, Cynthia Xiong, Zhongzhi Xu

**Affiliations:** 1 Centre for Suicide Research and Prevention, The University of Hong Kong, Hong Kong, China; judyhe@hku.hk (L.H.); chicoxyc@hku.hk (Y.X.); evanchy@hku.hk (E.C.); rence@hku.hk (F.C.); chowjt@hku.hk (J.C.); fjerry@hku.hk (J.F.); christhc@hku.hk (C.T.); cynxiong@hku.hk (C.X.); zhongzhi@hku.hk (Z.X.); 2 Department of Social Work and Social Administration, The University of Hong Kong, Hong Kong, China; flawhk@hku.hk; 3 Youth and Community Services, Caritas Hong Kong, Hong Kong, China; chancharlie@caritassws.org.hk (W.-L.C.); shirley.chow@openup.hk (S.C.); winnie.ng@openup.hk (W.M.N.); 4 Department of Psychology, The University of Hong Kong, Hong Kong, China; shaunlyn@hku.hk; 5 Head Office, Hong Kong Children and Youth Services, Hong Kong, China; swsc@hkcys.org.hk; 6 Department of Social Work, The Chinese University of Hong Kong, Hong Kong, China; qcheng@cuhk.edu.hk; 7 Youth Service, St. James’ Settlement, Hong Kong, China; sh.cheng@sjs.org.hk; 8 Headquarter, The Hong Kong Federation of Youth Groups, Hong Kong, China; siuman.hsu@hkfyg.org.hk; 9 IT Unit, The Hong Kong Federation of Youth Groups, Hong Kong, China; billie.lo@hkfyg.org.hk; 10 Jockey Club Online Youth Emotional Support, The Boys’ and Girls’ Clubs Association of Hong Kong, Hong Kong, China; workmiu@gmail.com; 11 Independent Researcher, Hong Kong, China; ykngai@gmail.com

**Keywords:** youth suicide prevention, crisis intervention, online emotional support service, online text-based counselling, Artificial Intelligence

## Abstract

We present the opportunities and challenges of Open Up, a free, 24/7 online text-based counselling service to support youth in Hong Kong. The number of youths served more than doubled within the first three years since its inception in 2018 in response to increasing youth suicidality and mental health needs. Good practice models are being developed in order to sustain and further scale up the service. We discuss the structure of the operation, usage pattern and its effectiveness, the use of AI to improve users experience, and the role of volunteer in the operation. We also present the challenges in further enhancing the operation, calling for more research, especially on the identification of the optimal number of users that can be concurrently served by a counsellor, the effective approach to respond to a small percentage of repeated users who has taken up a disproportional volume of service, and the way to optimize the use of big data analytics and AI technology to enhance the service. These advancements will benefit not only Open Up but also similar services across the globe.

## 1. Introduction

Suicide is a major global public health challenge [1]. It is one of the leading causes of death among youth in US [2] and around the world [1]. In Hong Kong, suicide is the most common cause of death for young people aged 15 to 24. Despite an overall reduction in suicide rate, the rate among youths has continued to demonstrate an upward trend [3].

Increased access to digital technologies has significantly changed youth’s means of communication. Social media, including text messaging, has become their primary medium of communication [4,5]. Its accessibility affords online services an effective way to reach at-risk youths, especially those are accustomed to this mode of communication. Adolescents and young adults generally prefer text messaging instead of phone calls, emails, and other social media for a wide variety of situations [6,7]. Compared to telephone hotline services, youths prefer text messaging as it reduces chances of being overheard and provides more privacy [8]. The anonymity of online services may also enhance young peoples’ sense of comfort and autonomy, which in turn facilitate self-disclosure [9]. The provision of anonymous text messaging services through social media or web portals appears to facilitate help-seeking among youths [10,11,12].

Given these developments in mode of communication, providing emotional support and suicide prevention services to youth through text-based online counselling services has become an emerging trend [13]. The advent of COVID-19 also furthered youths’ reliance and demand for online counselling services [14,15].

Online counselling services have been found to be effective in increasing young users’ resilience, enable timely identification and management of psychological issues, and reduce suicidal ideation [16]. Prime examples of nationwide services of this nature include Crisis Text Line, which has served over 6.1 million users since 2013 in the US, UK, Canada, and Ireland [17], and Lifeline Australia which accumulates over 93,000 text-based conversations each year [18]. In Hong Kong, responding to the alarming trends in youth suicide, Open Up, a 24/7 free text-based online emotional support service, was launched in October of 2018 [19].

### 1.1. Background

Open Up (www.openup.hk, archived on 13 December 2021) enables people aged 11–35 to chat with social workers or trained volunteers (together referred to as “counsellors”) anonymously. It can be accessed anywhere and anytime for free through various channels, including SMS, WhatsApp, Facebook Messenger, and the official web portal. 

Sponsored by The Hong Kong Jockey Club Charities Trust, Open Up is organized by one research centre—The Hong Kong Jockey Club Centre for Suicide Research and Prevention at The University of Hong Kong, and five of the most well-established local NGOs -Caritas Hong Kong, Hong Kong Children and Youth Services, The Boys’ and Girls’ Clubs Association of Hong Kong, The Hong Kong Federation of Youth Groups, and St. James’ Settlement.

**Figure 1:**
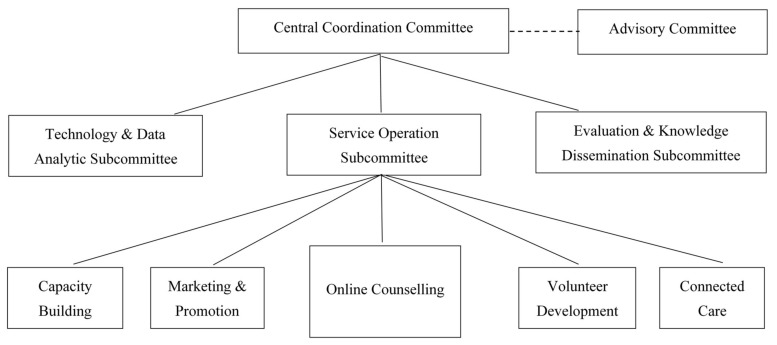
Open Up’s operation architecture and governance infrastructure.

Figure 1 shows Open Up’s operation architecture and governance infrastructure. The Central Coordination Committee (CCC) has overall coordination, monitoring, supervision and advocacy responsibilities of the service. Reporting to CCC are three subcommittees: the Service Operation Subcommittee, the Technology and Data Analytic Subcommittee, and the Evaluation and Knowledge Dissemination Subcommittee.

The Service Operation Subcommittee is responsible for the 24/7 online service operation, volunteer development, training and capacity building, marketing, and connected care follow-up services. The Technology and Data Analytic Subcommittee develops the IT operation systems, offers technology support, and provides data analysis dashboards. The Evaluation and Knowledge Dissemination Subcommittee is responsible for research, evaluation, and knowledge dissemination. Data are processed and analysed using mixed methods approaches, including both human annotation as well as machine learning and neural network modelling. The core aims of the subcommittee are to coordinate, identify, and disseminate good practices.

The Advisory Committee consists of local and international advisors from the government, academia, technology, and education sectors. It provides professional advice to ensure that Open Up meets the needs of at-risk youth. It also solicits support from key stakeholders in the community and holds regular meetings with the CCC.

Dinakar and colleagues reported using real time topic modelling and visualization for Crisis Text Line [20]. Open Up takes a step further to develop six Smart Modules that, employing AI and natural language processing (NLP) technologies, provides (i) real-time topic classification, (ii) risk assessment, (iii) practices reminders, (iv) recommendation for referrals or standardized responses (v) user segmentation, and (vi) big data analytics platform.

The Topic Classification Module was developed to identify and classify discussion topics and provide real-time visualisation to counsellors. The Risk Assessment Module assesses users’ suicidal risks using NLP and determines their risk level as low, medium, high, or crisis using a specific scoring system that factors in information pertinent to suicide ideation, plan, means, action, functioning, social support, and use of health support services. Users’ risk level is continuously assessed from one conversation block to another, and the conversation interface will display a visual red flag when the user displays high or crisis risk levels. Counsellors can also modify the level of risk as the conversation progresses. By displaying users’ risk levels, the module actively assists counsellors to determine the best course of action. The Stage Reminder Module reminds counsellors to consider key steps specified in the service protocol. These steps include safety checks, providing empathy, identifying core problems, exploring possibilities, and plans for follow-up. These steps are automatically checked when the system detects related key words in the conversation. This module provides guidelines for counsellors to structure and monitor their conversation progress, which is especially useful for novice counsellors. The Practices Recommendation Module provides information of relevant offline resources and recommended standardized (but modifiable) responses associated with on-going topics to counsellors in real time. For instance, when a user talks about the frustration of re-integration into the community after recovering from depression, information about community centres for mental wellness and suggested replies related to depression recovery are provided to the counsellor. The counsellor is free to adopt, adapt, or ignore these suggestions. This module creates a supportive work environment to counsellors and further improves service efficiency. The User Segmentation Module automatically stratifies users according to their similarities across various dimensions, such as age, gender, and concerned topics. This generates valuable information for research and service evaluation. Finally, the Big Data Analytics Platform visualizes analytics results of various aspects, including topics of concern, risk levels, and emerging keywords. The platform gives the project team a real-time overview of the user’s needs, user experiences, and service quality, which are crucial for service evaluation and improvement.

### 1.2. Current Study

As Open Up enters its fourth year of operation, this paper reviews its role and effectiveness in providing emotional support for youths through examining the service’s data and performance. We also present the innovative solutions Open Up developed to tackle challenges that arose, and conclude with insights on how to further advance, scale up, and sustain this type of text-based online counselling service.

## 2. Materials and Method

### 2.1. Data

Open Up service data including chat history and activity logs from users and counselors were recorded and stored in the Open Up platform in real-time. The data are regularly extracted for trend and thematic analyses. In this study, we extracted the data generated from October 2018 to June 2021 for investigation. A total of 29,400 users with 81,654 chats were included.

### 2.2. Descriptive Analysis

Data aggregation and data mining were used to churn out the historical data of the first three years of Open Up. We examined the following three aspects of the service: (1) Traffic and capacity. The 7-day moving average of the valid cases was used to present the overall trend of service capacity; (2) de-escalation of risk. We extracted the highest risk and the exit risk levels in each session and tabulated the risk level changes, which was used as a proxy of effectiveness; (3) topics of concerns presented by users. Multi-labeled topics detected by keywords/phrases and topic models with real-time modification made by counsellors were used to represent the main issues of concern of users. We counted the number and percentage of every topic.

## 3. Results 

### 3.1. Service Capacity

**Figure 2:**
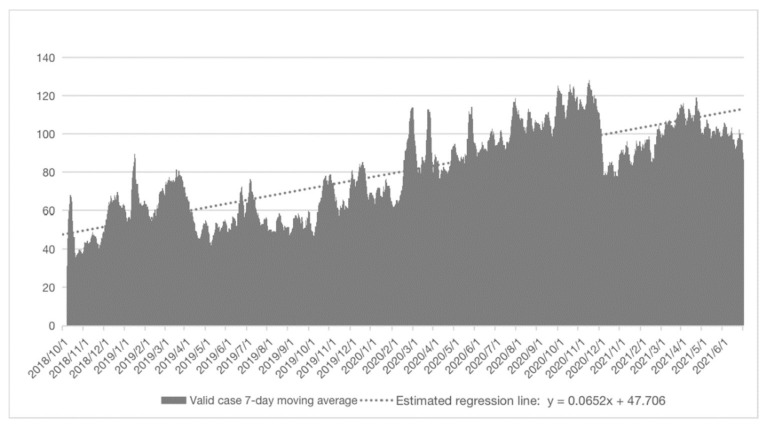
Number of valid cases by 7-day moving average from October 2018 to June 2021.

From Oct 2018 to June 2021, Open Up served a total of 81,654 valid sessions. A “valid session” is one that contains four or more message exchanges between the user and the counsellor. The average chat time of valid sessions was 67.2 min (SD = 58.5). The number of valid sessions increased gradually from an average of 43 per day in October 2018 to an average of 97 per day in June 2021 (Figure 2).

### 3.2. User Satisfaction

Through the voluntary post-service evaluation survey, which had a response rate of 17.4% (*n* = 81,654), 81.5% (*n* = 13,244) of the users found the service helpful and 85.4% (*n* = 12,688) were more likely to seek help in the future. Of those who were provided with community service information during the session, 79.0% (*n* = 5015) of users found the information helpful. Importantly, 45.5% (*n* = 11,917) of the users had never sought help from others about the issue they discussed in Open Up. In addition, as there is premature departure among the users, it is important to build up empathetic support in a timely manner [21].

### 3.3. De-Escalation of Risk

Counsellors would assign and adjust the risk level of each user throughout the session based on the perceived level of suicide risk. As shown in Table 1, of the valid sessions, 2.1% were categorised as crisis (i.e., the highest risk level), 1.5% as high, 14.8% as medium, and 81.6% as low risk levels. Based on the changes from the highest risk level during the session to the risk level when the session ended (“exit risk level”), Open Up lowered 85.3% of the high risk and crisis cases’ suicide risk levels during the intervention.

### 3.4. Returning Users

While some users are satisfied with the one-off counselling session, some expressed the need for longer interventions to address their problems and have used the service repeatedly. As reported in Table 2, approximately 34.5% of the users sought Open Up services multiple times. Meanwhile, 0.5% of the users visited Open Up for more than 50 times and contributed to 21.3% of the valid sessions. This not only challenges service capacity, but also highlights the need for designated care to meet these service-users’ needs and provide effective follow-up services.

### 3.5. Topics of Concern

**Figure 3:**
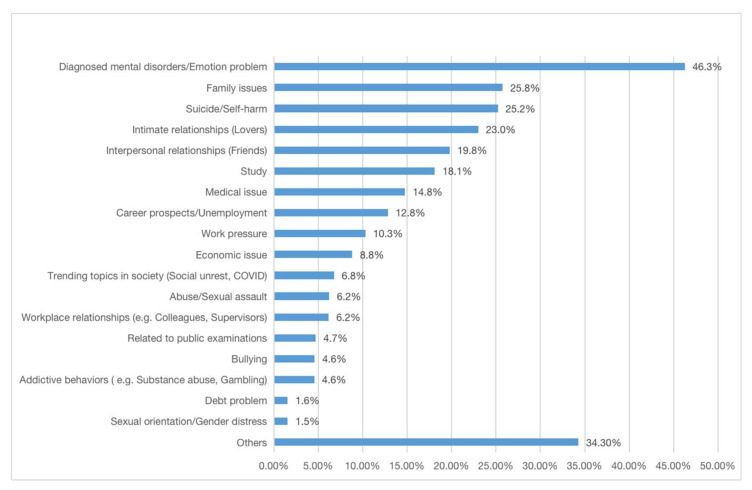
The topic distribution of Open Up users from Oct 2018 to June 2021. Note: The module is under improvement with machine learning.

Figure 3 reports the major topics identified through real-time analysis of keywords. In the review period, the most commonly discussed topics were mental health/emotion issues, family, intimate relationship, interpersonal relationship, study, medical issue, career prospects, and work pressure. These concerns are similar to the telephone hotline services in the city (e.g., Suicide Prevention Services) [22].

## 4. Discussion

The number of daily cases served by Open Up has doubled in 32 months, indicating an increasing demand for the service. Thus far, Open Up enjoys high user satisfaction and appears to be effective in de-escalating suicide risk levels. Below, we discuss a number of identified challenges and their corresponding innovative solutions.

### 4.1. Challenges

**Difficulties and Dropouts in Single-session Online Counselling**. Based on the existing data, the majority of Open Up users (65.5%) accessed the service only once, suggesting that counsellors have to strive to meet their service goals in a single session. This poses a challenge to the counsellor to establish rapport, assess needs, reduce risk, and relieve distress within a short period of time. Compared to verbal communication, text exchanges entail a time lag, which, together with the lack of non-verbal feedback and contextual clues, can also give rise to communication barriers and negatively impact rapport building [23,24]. Similar to tradition single-session therapy [25], these characteristics of synchronous online text-based counselling require the skill to carefully, yet rapidly, cultivate a therapeutic relationship with the user, identify workable presenting problems, develop a realistic yet meaningful goal, and provide appropriate follow-up and referral information.

However, unlike other modalities, counsellors in online text-based counselling have fewer cues to work with. The termination point of each text-based session can be ambiguous and unpredictable [21]. Additionally, predictors of adherence and dropout in face-to-face psychological treatments have been largely inconsistent across contexts and modalities [26,27]. This issue is compounded by the anonymity of text-based counseling services—as information about the user’s identity is limited, there are additional difficulties in knowing whether the user is telling the truth, or in ascertaining their safety. If the user chooses not to complete the post-session survey, there is limited information available with which to evaluate the session and quality of service. For repeated and even abusive users, it is difficult if not impossible to identify them before they enter the chat; additional time and sources of information is needed if the counsellor wishes to refer to previous session notes in order to make meaningful progress with the user.

Uncovering the reasons behind repeated- or single-use of online youth counselling thus remains as a research gap. Filling such knowledge gaps will likely afford more efficient planning of services.

**Rebalancing of Staff and Volunteers for Long Term Sustainability**. The doubling of Open Up’s daily traffic in less than three years of operation has strained existing staff’s capacity. Currently, the distribution of caseload is approximately 70% on paid staff and 30% on volunteers. A longer-term solution to the rise in demand is to increase the reliance of volunteers, which is a core objective of the project since its inception. To achieve long term sustainability, in addition to engaging more volunteers, paid staff can also be encouraged to become full-time trainers and supervisors. This lowers operation costs, facilitates service expansions, and extends the Open Up service to benefit volunteers. The recruitment and retention of the volunteers are crucial for the future sustainability of the service, especially if the current funding level cannot be maintained in the long run. The roles of paid staff and volunteers with different skill levels in service delivery have yet to be optimally deployed. As the experienced paid staff is very instrumental to develop quality volunteer service for the system, the turnover rate of paid staff needs to be monitored to enhance capacity building and knowledge transfer of online emotional support.

### 4.2. Innovative Solutions

**Beyond One-Time Service: Connected Care**. To respond to the needs of returning users, Open Up has a “Connected Care” ecosystem that links users to existing online and offline services. When deemed appropriate by the counsellor and the supervisor, a service user would be referred to online and offline follow-up services that are provided by various strategic partners, including those offered by the five partnering NGOs, as well as other programs.

The program is developing a Connected Care Platform, which is designed to allow both the Open Up team and the receiving service to track referral service use and user progress. This platform aims to minimize the gap between the two services and to ensure that the user is provided with ample opportunities to consider the recommended referral service. Post-service surveys, referral case statistics, and service providers performances are monitored closely to improve service quality.

One of the follow-up options is in-house online service called Thematic Care. This service is designed for youth who face challenges in a specific area (i.e., theme/module-based) but prefers online text-based counselling over face-to-face services, or those who need a transition period from online to offline services. Examples of modules include specific mental health related issues, interpersonal relationships, and career and life planning. To initiate the service, a counsellor would make an assessment and refer those in need to the dedicated in-house team. Designated counsellors with expertise in the theme or issue in question would then provide 4 to 6 scheduled online appointments. When appropriate, these sessions also serve to help users identify core issues associated with their risk of self-harm/suicide, develop a care plan and coping strategies, and prepare them to receive offline help (e.g., via Connected Care) if needed. The Evaluation and Knowledge Dissemination Subcommittee regularly review and evaluate the effectiveness of the Connected Care referral system and the in-house theme-based services. The goal is to help users identify the most appropriate service, streamline the traffic of the regular 24/7 Open Up chat service and enhance users’ experiences and its effectiveness. Collaboration should be explored and developed with other organizations which provides similar services.

**Smart Modules Empowered by Artificial Intelligence**. As reported in the Introduction, Open Up developed a Smart Module system that, among other things, provides real-time classification of topics and risk assessment. Counsellors can adjust the module-generated topics and risk level. Such adjustments would help further improve the sensitivity and specificity of the system. The obtained information provides important insights to understand help-seeking trends, prepare service operations, and enhance trainings of counsellors. Monitoring of trends and risk levels is performed on a regular basis by various subcommittees. At times, information about emerging trends and needs are shared with other stakeholders as a means to improve community preparedness. Collectively, these smart modules have shown to be particularly helpful in supporting volunteers and new staff counsellors by providing them with key information, visual tools, and reminders throughout each session.

**Concurrent Chats to Scale Up Service Capacity**. In individual face-to-face counselling, sessions are conducted between a client and a counsellor. However, online text-based counselling makes it possible for counsellors to handle multiple chats concurrently. As the service demand picks up, Open Up counsellors engage more than one user at the same time, especially during peak hours. When handling high risk and crisis cases, counsellors would focus on only one case at a time.

While the idea of concurrent chats is encouraging as it doubles or even triples the service capacity, its influence on counsellors, users, and the overall service quality needs close examination. As dividing attention can reduce performance, with potential moderators such as the time of a day, fatigue, and sleep quality [28,29], it is unclear how the division of attention affects counsellors in handling sessions effectively and efficiently. As there are individual differences in the lapse of attention [30], it remains a question whether there is a one-size-fits-all threshold of the number of concurrent chats for counsellors, as they have different skillsets and handle different types of cases at a different time of a day. The project team is diving deep into the service data as well as counsellors’ and users’ feedback in the hope to address the question.

**Engaging the Wider Community: Volunteers, Gatekeepers, and Mental Health Professionals**. In addition to providing direct services and referral to users, Open Up also aims to train dedicated volunteers, recruit suicide prevention gatekeepers from parents, teachers, and peers to provide immediate emotional support to vulnerable youth, and engage local and global mental health professionals through the sharing of practice model and experience. To date, Open Up has successfully trained 488 volunteers, 834 gatekeepers, and promoted online-counselling experience and knowledge to both professionals and the general public through more than 50 sharing sessions.

The ongoing effort involves conducting online counselling skills with practicum trainings for volunteers. Volunteers are ensured adequate support by matching each of them with two designated social workers as mentors who monitor their performance and provide emotional support.

Gatekeeper trainings aim to equip friends, classmates, families, co-workers, educators, and care-givers with knowledge and skills to identify and support youth with suicide risk. These trainings mainly take place at secondary schools and tertiary institutes in the forms of talks and experiential games. Skills on identification of warning signs, including verbal, behavioural, and situational cues are shared. A graduate course on social media and online counselling has been co-created with a local university to raise the interest and enhance expertise in the field.

Taken together, online counselling has become an emerging tool in helping young people in need. Furthermore, there have also been increasing use of technology to help minimize the input of and burden on human counsellors. The use of big analytic and AI technology using text, voice and facial detection are emerging and promising trends. The real challenge is how to integrate them so as to provide appropriate and timely care that makes use of online and possibly offline support effectively and efficiently.

Compared to other well-established services such as the US Crisis Text Line, Open Up has experienced a steep 32-month learning curve and identified a few challenges in online emotional support. As the demand is growing, the service continues to seek ways to expand service delivery, develop innovative measures, and implement good practices. Long-term sustainability of Open Up requires continuous funding support, other service operators, and potential community partners who champion Open Up’s mission. Open Up also aims to work towards becoming an inclusive community, in which like-minded individuals not only directly provide support to youths in need, but also co-operates and supports one another. Volunteers training and retention become a crucial element for its operation. Hence, Open Up prioritises the creation of a friendly and supportive work environment where staff and volunteers can establish lasting friendships. As suggested by numerous studies [31,32], volunteers can remain motivated and committed if they find high levels of satisfaction in the work and if their sense of calling aligns with the mission of the services. These, in turn, can help lower volunteer attrition and increase their stake in the project.

## 5. Conclusions

Text-based online counselling is a novel and emerging form of crisis intervention. It is particularly relevant in the “new normal” under COVID-19, when diversity of service modality is needed to meet the rapid changes in physical distancing restrictions. Furthermore, how to provide relevant and effective support to young people in this digital era remains a challenge worth tackling. Open Up is committed for continuous learning and improvement. We will also continue to share our first-hand experiences to mental health care professionals locally and aboard. Our service strives to meet the Sustainable Development Goal of not leaving anyone behind [33]. To achieve this lofty goal, we believe Open Up needs to continue to identify and empirically test creative, effective, and sustainable solutions for continuous improvement and enhancement.

## Figures and Tables

**Table 1:** Risk level changes during the intervention (*n* = 81,654).

Highest Risk Level	Exit Risk Level			
	Low	Med	High	Crisis
Crisis (1687)	681 (40.4%)	713 (42.3%)	110 (6.5%)	183 (10.8%)
High (1210)	397 (32.8%)	571 (47.2%)	242 (20.0%)	0
Med (12,103)	6122 (50.6%)	5981 (49.4%)	0	0
Low (66,654)	66,654 (100.0%)	0	0	0

**Table 2:** Percentage of users and cases by number of valid visits in the study period.

Valid Visits	User Percentage (*n* = 29,400)	Case Percentage (*n* = 81,654)
1 time	65.5%	23.6%
2 times	16.1%	11.6%
3–5 times	12.0%	15.5%
6–10 times	3.3%	8.8%
11–20 times	1.6%	8.0%
21–50 times	1.0%	11.2%
over 50 times	0.5%	21.3%

Note: Identified by unique identifiers: (1) IP addresses via web portal; (2) unique number via SMS, Facebook Messenger and WhatsApp.

## Data Availability

Due to the nature of this research, participants of this study did not agree for their data to be shared publicly, so supporting data is not available.
